# Characterization of a Versatile Plant Growth-Promoting Rhizobacterium *Pseudomonas mediterranea* Strain S58

**DOI:** 10.3390/microorganisms8030334

**Published:** 2020-02-27

**Authors:** Yilin Gu, Jing Wang, Zhenyuan Xia, Hai-Lei Wei

**Affiliations:** 1 Key Laboratory of Microbial Resources Collection and Preservation, Ministry of Agriculture and Rural Affairs, Institute of Agricultural Resources and Regional Planning, Chinese Academy of Agricultural Sciences, Beijing 100081, China; guyilin@caas.cn (Y.G.); wangjing27@hotmail.com (J.W.); 2 Yunnan Academy of Tobacco Agricultural Science, Kunming 650021, China; zyxia@yntsti.com

**Keywords:** *Pseudomonas mediterranea*, biological control, cyclic lipopeptide, cell death

## Abstract

Plant growth-promoting rhizobacterial strain S58 was isolated from the tobacco rhizosphere. It showed strong antagonism against a battery of plant pathogenic fungi and bacteria, and controlled wheat sharp eyespot and tobacco wildfire diseases efficiently. Further tests showed that strain S58 solubilized organic phosphate and produced siderophore, protease, ammonia, and indole-3-acetic acid. In *Arabidopsis thaliana*, it promoted plant growth and changed root system architecture by restricting the growth of primary roots and increasing lateral root numbers. We relied on morphological, biochemical, physiological characteristics, and molecular phylogenic analysis to identify strain S58 as *Pseudomonas mediterranea*. The complete genome of strain S58 has a single circular chromosome of 6,150,838 bp with a 61.06% G+C content. The bacterial genome contained 5,312 predicted genes with an average length of 992.90 bp. A genome analysis suggested that *P. mediterranea* S58 was a rich cyclic lipopeptide (CLP)-producing strain that possessed seven non-ribosomal peptide gene clusters for CLP synthesis. Leaf inoculation of the bacterial culture and supernatants triggered cell death-like immunity in tobacco. Quantitative real-time PCR assays showed that the strain S58 induced the expression of pattern-triggered immunity and cell death marker genes, but not jasmonic acid marker genes. The results suggested that *P. mediterranea* S58 is a novel, versatile plant growth-promoting agent with multiple beneficial traits for plants.

## 1. Introduction

Beneficial microbes have received a great deal of attention as a sustainable alternative for improving plant health. *Pseudomonas* is a well-known plant growth-promoting rhizobacterial (PGPR) genus for its multifarious plant beneficial functions. The mechanisms of a class of *Pseudomonas* species, such as *P. fluorescens*, *P. protegens*, *P. chlororaphis*, and *P. putida*, have been studied intensively and used widely in agricultural applications [1,2]. The PGPR *Pseudomonas* strains exert positive effects on plants in many ways, such as direct antagonism against pathogens, induction of plant resistance and immunity, alteration of plant growth morphology, toleration of environmental stress, and utilization of minerals [3,4]. Cyclic lipopeptides (CLPs) and polyketides that are synthesized by non-ribosomal peptide synthetases (NRPS) and by polyketide synthases (PKS), respectively, are major antimicrobial secondary metabolites produced by PGPR *Pseudomonas* [5,6,7]. *Pseudomonas* isolates, such as *P. kilonensis* F113, *P. protegens* Pf-5, and *P. fluorescens* SBW25 and 2P24 produced 2,4-diacetylphloroglucinol (DAPG), phenazines, pyoluteorin, pyrrolnitrin, viscosin, orfamide A, and amphisin to inhibit a broad range of plant pathogens directly [8,9,10,11]. In addition to secondary metabolite-mediated, antagonistic effects on pathogens, the biological control activity of *Pseudomonas* strains is also linked to the induction of induced systemic resistance (ISR) and siderophore-mediated competition for iron [12]. *P. simiae* WCS417 and other strains colonized plant root systems and induced ISR to prime host immunity, which provided enhanced protection against a broad spectrum of plant pathogens [12]. Some other *Pseudomonas* bacteria promoted host plant growth by solubilizing phosphorus, fixing atmospheric nitrogen, and synthesizing phytohormones [13,14,15,16]. Therefore, the identification of PGPR and biological control agents (BCAs) from different matrices, mainly from the plant rhizosphere, is a significant research direction for improving plant health and stress resistance.

*P. mediterranea*, along with its closely related species *P. corrugata*, are recognized as two causal agents isolated from tomato pith necrosis [17,18]. Actually, *P. mediterranea* and *P. corrugata* are rather ubiquitous and richly present in the plant rhizosphere, and they are characterized as having a high metabolic versatility to promote plant health [17]. Many strains of the two species, especially *P. corrugata*, have been used widely as biological control agents in the management of fungal and bacterial diseases of plants. *P. corrugata* 2140, which is isolated from the wheat rhizosphere, reduced wheat take-all disease, and the Pythium root rot in sugarbeet [19,20]. The *P. corrugata* CFBP 5454 inhibited the growth of plant pathogenic fungi and bacteria by CLP production, which is controlled by a LuxR transcriptional regulator and the quorum sensing system [21,22]. In comparison, *P. mediterranea* has been used rarely as PGPR, but some strains such as CFBP 5447^T^ have been well-studied to efficiently synthesize medium-chain-length polyhydroxyalkanoate elastomers (mcl-PHA) and extracellular products [23].

In this study, we reported a new isolate of *P. mediterranea* from the tobacco rhizosphere. The strain S58 exhibited versatile plant growth-promoting traits, such as direct antagonism against a class of plant pathogens, solubilization of organic phosphate, production of siderophores, protease, ammonia, and indole-3-acetic acid, and the induction of plant immunity. Genome sequencing and comparative genomic analysis revealed that it possessed an arsenal of secondary metabolites. The data we obtained will increase the knowledge of the plant growth-promoting potential in *P. mediterranea*, and they will allow for the further study of adaptation mechanisms of *P. mediterranea* S58.

## 2. Materials and Methods

### 2.1. Bacterial Isolation and Identification

Soil samples were collected from the tobacco rhizosphere in Kunming, China. Two grams of soil sample was suspended in 18 mL sterile distilled water and spread on Lysogeny broth (LB) media for bacterial isolation. The individual colonies with different colors or morphology were selected for further purification and confirmation. The bacterial cells were stained with 1% (w/v) phosphotungstic acid first and then visualized with a transmission electron microscope (Hitachi-H600, Japan). The physiological and biochemical characteristics were determined by GEN III MicroPlates™ (Biolog, Hayward, CA, USA), according to the manufacturer’s protocol. The utilization pattern was monitored on an OmniLog^®^ Incubator/Reader (Biolog, Hayward, CA, USA). This assay was repeated twice. Oligonucleotide primers 27F (5′-GAGAGTTTGATCCTGGCTCAG-3′) and 1494R (5′-CTACGGCTACCTTGTTACGA-3′) were used for 16S rDNA amplification, as described previously [24]. The PCR product was sequenced at GENEWIZ Ltd., China. 16S rDNA, *gyrB*, *rpoB*, and *rpoD* gene sequences were extracted from the PseudoMLSA Database (http://microbiologia.uib.es/bioinformatica/) and used for a phylogenetic analysis [25]. The accession numbers of *gyrB, rpoB*, and *rpoD* in strain S58 are HG325842, HG325844, HG325843, respectively. MEGA X was used for multiple alignments, and the neighbor-joining and p-distance methods were used to construct phylogenetic trees [26].

### 2.2. Antagonistic Test

**Figure 1:**
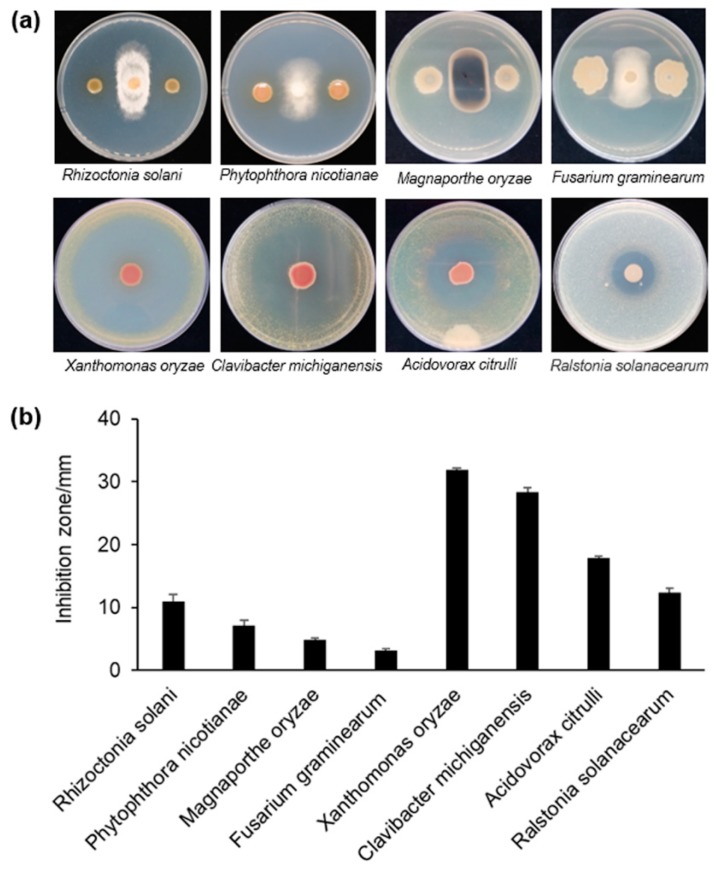
In vitro antimicrobial activity of strain S58 against different phytopathogens. **(a)** Four plant fungal pathogens (upper) and four plant bacterial pathogens were chosen to test the antimicrobial activity of strain S58; **(b)** Antimicrobial activity was estimated by measuring the diameter (mm) of the clear zone of growth inhibition. Results are expressed as the mean and standard deviation.

Eight plant pathogens that included fungi and bacteria (Figure 1) were chosen to test the antagonistic capacity of strain S58. First, fresh fungal disks were inoculated onto the Potato Dextrose Agar (PDA) plate center, and 5 µL of saturated strain S58 culture (ca. OD_600_ =1.8) was dotted along the two sides of the fungal disks at a distance of 30 mm. The inhibitory zones were measured after incubating at 25 °C for 3–4 d. For antibacterial tests, 5 mL of saturated pathogenic bacteria were mixed with 45 mL LB media that were pre-melted and incubated at 50 °C and poured onto plates. Five µL of saturated strain S58 culture was dropped onto the plate center. The inhibitory zones were measured after incubating at 28 °C for 2–3 d. All of the tests were performed in triplicate.

### 2.3. Disease Control Assay

For fungal disease, wheat (cv. Mingxian 169) seeds were disinfected with 5% sodium hypochlorite for 5 min and soaked in LB culture of strain S58 at 5 × 10^8^ CFU/mL with 0.02% Silwet L-77 for 30 min. Fresh LB medium was used as mock. Ten seeds each were planted in 14 mL Falcon™ tubes with sterilized soil. A 15 mm-diameter fungal disk of *Rhizoctonia solani* on PDA was placed on the top of the soil. The mock covered with fungal disks was referred to as disease control, and the mock that was covered with fresh PDA disks was referred to as the healthy control. It was maintained at room temperature for 7 d, and then they were dug out for disease measurements.

For bacterial disease, four- week-old *Nicotiana benthamiana* was used for tobacco wildfire assays. A fresh lawn of strain S58 was scraped from overnight plates with a sterile loop and suspended in 10 mM MgCl_2_. The suspension was sprayed on the leaves of *N. benthamiana* at 5 × 10^8^ CFU/mL with 0.02% Silwet L-77 and kept in the chamber for 24 h. A buffer of 10 mM MgCl_2_ was used as mock. A fresh pathogen suspension of *Pseudomonas syringae* pv. *tabaci* was then sprayed at 10^7^ CFU/mL with 0.02% Silwet L-77 on the leaves that were pre-inoculated with strain S58. The mock sprayed with the pathogen suspension was referred to as disease control, and the mock that was sprayed with 10 mM MgCl_2_ again was referred to as the healthy control. Inoculated plants were maintained at 70% humidity at 22°C with 16 h of light and 8 h of darkness. The symptoms were recorded at seven days post-inoculation. The assays were repeated twice.

### 2.4. Identification of Plant Growth-Promoting Traits

Strain S58 was characterized by typical plant growth-promoting traits qualitatively. Phosphate solubilization tests were performed [27], and the synthesis of siderophores was analyzed [28]. Enzymatic activity of protease, amylase, and cellulose was measured by the clearing zone technique [29]. Production of ammonia and indole-3-acetic acid (IAA) was detected as usual [30]. For the IAA assay, 100 μg/mL IAA was used as positive control.

*Arabidopsis thaliana* Col-0 was used to test the plant growth-promoting capacity. First, the seeds were surface-sterilized and then sown on Murashige and Skoog (MS) agar plates for stratification for 2 d at 4 °C [31]. The seed plates were moved into a plant growth chamber for vertical incubation at 21 °C with 16/8 h photoperiod. The bacterial suspension (250 µL, 10^8^ cfu/mL) was applied to the agar 5 cm below the seedlings. The same volume of MgSO_4_ (10 mM) was applied as a mock treatment. Primer root length and lateral root numbers were determined after 10 d. All of the tests were repeated three times.

### 2.5. Genome Sequencing and Comparative Genomic Analysis

Purified genomic DNA was used for library construction and sequencing by GENEWIZ Ltd., China. The complete genome was sequenced using an Illumina Hiseq and PacBio RSII platform, and then it was assembled using HGAP software. The coding genes were annotated using the National Center for Biotechnology Information (NCBI) nr database by Diamond. The functional annotation of genes was based mainly on protein sequence alignment using the databases NR, swiss-prot, Pfam, EggNOG, GO, and KEGG. The comparative analysis of the chromosomes between strain S58 and related *Pseudomonas* species was performed using GenomeMatcher software (http://www.ige.tohoku.ac.jp/joho/gmProject/gmhomeJP.html) and the bl2seq program [32]. The ANI calculator (www.ezbiocloud.net/tools/ani) was used to calculate the average nucleotide identity (ANI) [33]. A Ring Image Generator (BRIG) was used for genome comparison [34]. Secondary metabolite gene clusters were predicted using antiSMASH 5.0, a web-based analysis platform (http://antismash.secondarymetabolites.org/) [35]. The Bacterial Pan-Genome Analysis (BPGA) pipeline was used for the pan-genome analyses [36]. The cut-off value was set up to 50% to obtain the pan and core genomes. Venn diagrams were drawn using OrthoVenn2, which is a web server for the comparison and analysis of whole-genome orthologous clusters [37].

### 2.6. Assay of Cell Death

To test cell death elicitation, the bacterial suspension of strain S58 was infiltrated into the leaves of four-wk-old *N. benthamiana*, *Nicotiana tabacum*, and tomato (*Lycopersicon esculentum* cv. Moneymaker) at 10^7^ CFU/mL, 5 × 10^7^ CFU/mL, 10^8^ CFU/mL, 5 × 10^8^ CFU/mL, respectively. Inoculated plants were maintained at 70% humidity at 22°C with 16/8 h photoperiod for visualized cell death. To test the ability of the supernatant of strain S58 to trigger cell death, 100 mL saturated LB culture of strain S58 was centrifuged at 8000 rpm for 20 min at 4 °C. The supernatant was collected after being filtered through a 0.2 µm membrane. The filtrates were lyophilized and then extracted with methanol two times. Finally, it was dissolved in 2 mL methanol at 50 times concentration. Leaves of *N. benthamiana* were inoculated with this extract. To determine the thresh-hold concentration of cell death, the 50 times extract was diluted with methanol to 10 times and five times concentration. Leaves were photographed at 2 d post-inoculation. Each experiment was repeated twice.

### 2.7. Quantitative Reverse Transcription PCR

Fresh bacterial cells of strain S58 were suspended in 10 mM MgCl_2_ and infiltrated into the leaves of *N. benthamiana* at 10^8^ CFU/mL. After 12 h, total RNA of the inoculation area was isolated using the Tri-Reagent (Omega Bio-tek, USA) and treated with RNase-free DNase (Tiangen, China). The RNA integrity was determined by 1.2% agarose gel electrophoresis. Purified RNA (2 μg) was used to prepare cDNA using M-MLV reverse transcriptase (Tiangen, China) with random primers, according to the manufacturer’s instructions. qRT-PCR was performed in 96-well plates on an ABI QuantStudio6 Flex real-time PCR system (Applied Biosystems, USA). The reaction mix was performed using 5 μL of FastStart Universal SYBR Green Master (New England Biolabs, USA), 2 μL of 2 μM primer mix (final concentration at 0.4 μM), 2 μL of a diluted 1:10 cDNA, and water to complete a final volume of 10 μL. Cycling conditions were 95 °C for 10 min, and 40 cycles at 95 °C for 15 s, and 60 °C for 1 min. Primer sequences are shown in Table S1. The housekeeping gene EF1a was used as a reference. Fold changes in expression were calculated using the delta-delta Ct (ddCt) method as usual. Three biological and three technical replicates of the experiments were performed.

### 2.8. Statistical analysis

All of the experiments were performed in triplicate, and the mean and SD are shown. Tukey’s HSD test (*p* < 0.05) was used for statistical analysis of the results.

## 3. Results

### 3.1. Antagonism Against Phytopathogens

We screened a bench of bacterial isolates, in which strain S58 showed the broadest range in the antimicrobial spectrum. It inhibited the growth of fungi and bacteria, which included most of the pathogens that cause soil-borne diseases, such as *Rhizoctonia solani* that causes wheat sharp eyespot, *Phytophthora nicotianae* that causes tobacco black shank, *Fusarium graminearum* that causes corn stalk rot, and *Rastonia solanacearum* that causes bacterial wilt. S58 also inhibited pathogens that cause seed-borne diseases, such as *Xanthomonas oryzae* that causes rice leaves blight, *Clavibacter michiganensis* that causes tomato bacterial canker, and *Acidovorax citrulli* that causes cucurbit bacterial fruit blotch. Pathogens of aerial-borne diseases, such as *Magnaporthe oryzae* that causes rice blight, was also inhibited (Figure 1a). Overall, strain S58 had stronger antagonistic capacity against phytobacteria than against pathogenic fungi based on quantitation of the inhibition zone (Figure 1a). It inhibited the growth of *X. oryzae* and *C. michiganensis*, especially, that had inhibition zones that extended up to 30 mm (Figure 1a). These results indicated that the strong, broad antagonism of strain S58 might result in potent biological control. The inhibition by this phenotype suggested that strain S58 produced strong diffusible antimicrobial metabolites.

### 3.2. Biological Control of Plant Diseases

**Figure 2:**
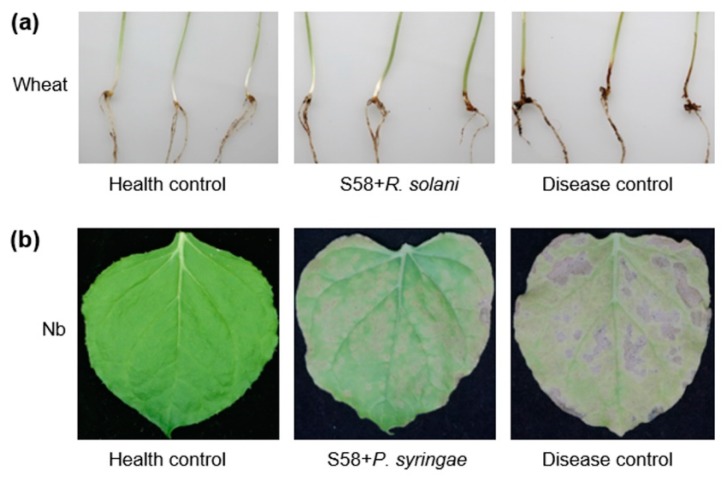
Disease management by strain S58. (**a**) Wheat sharp eyespot disease controlled by strain S58; (**b**) tobacco wildfire disease controlled by strain S58. The healthy control groups were treated with medium or buffer. S58+*R. solani* and S58+*P. syringae* represent that the plants were pretreated with strain S58 prior to being treated with the pathogens. Disease control groups were only treated with pathogens *R. solani* and *P. syringae*. Wheat represents wheat cv. Mingxian 169 and Nb represents *N. benthamiana*. The symptoms have been described in the text.

Given the notably inhibitory effect on plant pathogens, the biocontrol potential of strain S58 was determined in a pot experiment. At 7 dpi of planting, wheat seedings were dug to quantify black shank symptom. The group that served as the healthy control survived and were symptomless (Figure 2a). The disease control group treated with *R. solani* developed tan cortical rot and lesions on the roots and basal leaf sheaths (Figure 2a). The lesions were elliptic or “eye” shaped with a tan center surrounded by a dark brown margin and had an average length of 12.0±2.4 mm. Surprisingly, wheat seedings pre-soaked with strain S58 and then treated with *R. solina* had smaller lesions with an average length of 3.2±1.1 mm (Figure 2a). This demonstrated that strain S58 protected plants from *R. solani*, an important soil-borne disease.

Tobacco wildfire is a foliar disease that is caused by the plant pathogenic bacterium *Pseudomonas syringae* pv. *tabaci*. The healthy control group exhibited no symptoms, but the disease control group exhibited chlorotic halos and necrotic spots that coalesced and expanded irregularly (Figure 2b). Infected leaves became brown and distorted. Significantly, the leaves pre-treated with strain S58 only had small circular pale-green areas and did not develop chlorosis and necrosis (Figure 2b, Figure S1). Strain S58 inhibited the growth of a number of phytobacteria, but not *P. syringae* pv. *tabaci* (Figure S2). The results suggested that direct antagonism against pathogens was not the only way that the strain S58 controlled plant disease.

### 3.3. Plant-Growth Promoting Activities

**Figure 3:**
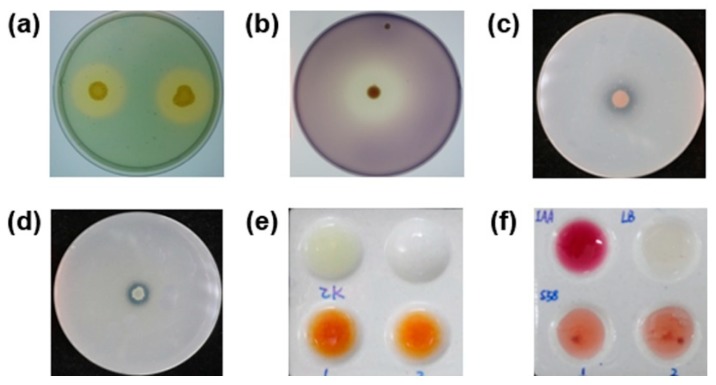
In vitro test of plant growth-promoting traits of strain S58. The transparent zone around the colonies are visualized due to **(a)** siderophore production, **(b)** amylase production, **(c)** protease production, and **(d)** phosphate solubilization. **(e)** Ammonia production is shown from the visualized brown, yellow color treated with strain S150 (lower two replicates) compared with the upper left water control. **(f)** Indole-3-acetic acid (IAA) production is shown from the visualized pink color treated with strain S58 (lower two replicates) compared with the upper left positive control (100 mg/mL IAA) and upper right negative control (LB medium).

We used selective media to test a series of plant growth-promoting traits qualitatively. The formation of clear hydrolytic halos around the colonies demonstrated that strain S58 produced siderophores, amylase, protease, and solubilized organic phosphorus (Figure 3abcd). The development of a brownish color indicated the production of ammonia (Figure 3e). The Salkowski reagent was used for the detection of IAA. The reagent treated with the bacterial culture of the strain S58 became dark pink, which suggested the formation of an IAA complex and a reduction of Fe^3+^ (Figure 3f).

**Figure 4:**
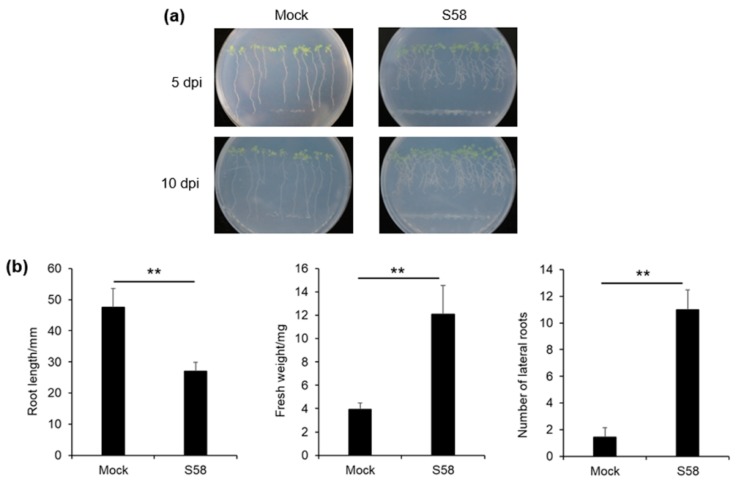
Effects of strain S58 on plant growth and root system architecture of *Arabidopsis thaliana* Col-0 seedlings. **(a)** The root architecture changed on the S58 plates at 5 dpi and 10 dpi; **(b)** Root length, fresh weight, and lateral roots were measured at 10 dpi. Stars indicate statistically significant differences (Tukey’s HSD test; *p* < 0.01).

The plant growth-promoting effect of strain S58 studied in *Arabidopsis thaliana* seedlings that grew vertically on agar-solidified medium, in which the seeds were challenged with strain S58. As time went by, the primary root length (PRL) of seedlings exposed to the strain S58 was reduced, and the lateral root number (LRN) increased (Figure 4a). After 10 d of cocultivation, the PRL in the mock control grew up to 5 cm, which was the distance of the inocula from the seeds (Figure 4b). PRL in the treatment with strain S58 was reduced by approximately 50% (Figure 4b). At the same time, strain S58-treated roots formed approximately 5.5-fold more LRN compared with the mock control, and the fresh weight increased three-fold also (Figure 4b). These results showed that S58 promoted plant growth and stimulated plant biomass production by affecting the architecture of the root system.

### 3.4. Identification of Strain S58

Strain S58 was characterized as a multi-functional beneficial bacterium, as shown above. We then made a comprehensive classification for it. The colonies of strain S58 on LB medium were round, wrinkled with a mucous texture, and often produced a diffuse yellowish non-fluorescent pigment. The growth on KB and under UV did not reveal a fluorescent pigment. The bacterial cells of strain S58 were rod-shaped, 0.4–1.2 μm in diameter, and 1.3–4.3 μm long. Only one flagellum at one polar was observed under a transmission electron microscope (Figure S3). From the results of the GEN III MicroPlates™ assay (Table S2), strain S58 had a very similar biochemical profile to *Pseudomonas mediterranea* [18], such as utilizing L-alanine, aminobutyrate, D-fructose, D-galactose, D-gluconate, D-glucose, L-glutamate, lactate, L-malate, D-mannitol, D-mannose, mucate, L-proline, quinate, D-saccharate, sucrose, D-trehalose, etc. and not utilizing D-arabitol, D-cellobiose, L-fucose, gentobiose, L-histidine, lactose, maltose, D-melibiose, D-raffinose, L-rhamnose, D-sorbitol, L-tartrate, tricarballylate, D-turanose, etc.

**Figure 5:**
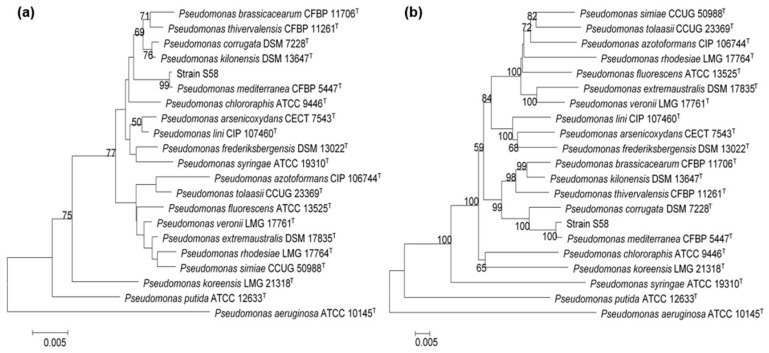
Phylogenetic tree showing the relationship of strain S58 with other *Pseudomonas* spp. type strains. (**a**) Phylogenetic analysis based on the 16S rRNA gene sequences; (**b**) Phylogenetic analysis based on four core housekeeping genes (*16S rRNA, gyrB, rpoB, rpoD*). *P. aeruginosa* ATCC 10145^T^ was used as an outgroup. Bootstrap values from 1000 replicates are indicated at the nodes.

To carry out a more accurate identification of the bacterium, we performed a phylogenic analysis of the 16S rRNA gene. The 16S rDNA sequence of strain S58 had 99.04% identity with that of *P. mediterranea* type strain CFBP 5447^T^. The phylogenic tree of 16S rRNA genes showed that strain S58 clustered with the type strain of *P. mediterranea* with a high bootstrap score (>99%) (Figure 5a). For an accurate determination, a multi-locus sequence analysis (MLSA) using the 16S rRNA gene along with the sequences of the housekeeping genes *rpoB*, *rpoD*, and *gyrB* was conducted. The results indicated that strain S58 had the same phylogenetic distribution as analyzed with the 16S rRNA gene (Figure 5b).

**Figure 6:**
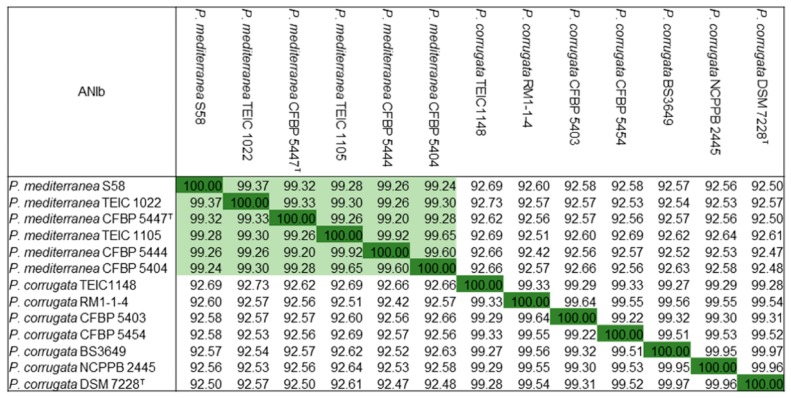
Average Nucleotide Identity based on BLAST (ANIb) for most of the genome-released strains of *P. mediterranea* and *P. corrugata*. Green shading indicates the same species of *P. mediterranea*.

*P. mediterranea* and *P. corrugata* are the most closely related species. To establish further the phylogenetic relationship of strain S58, we compared the available genomes of representative strains in the two species by calculating the average nucleotide identity (ANI) values. Strain S58 had higher ANI values >99% with *P. mediterranea* strains that included the type strain *P. mediterranea* CFBP 5447^T^. However, all *P. corrugata* strains showed ANI values <94%, which have been proposed for defining the boundary between species (Figure 6). In conclusion, morphological, physiological, and biochemical characteristics along with phylogenetic and ANI analyses revealed that strain S58 belonged to *P. mediterranea*. A culture of S58 was deposited in the China General Microorganism Culture Collection (CGMCC17043).

### 3.5. Comparative Genomic Analysis

**Figure 7:**
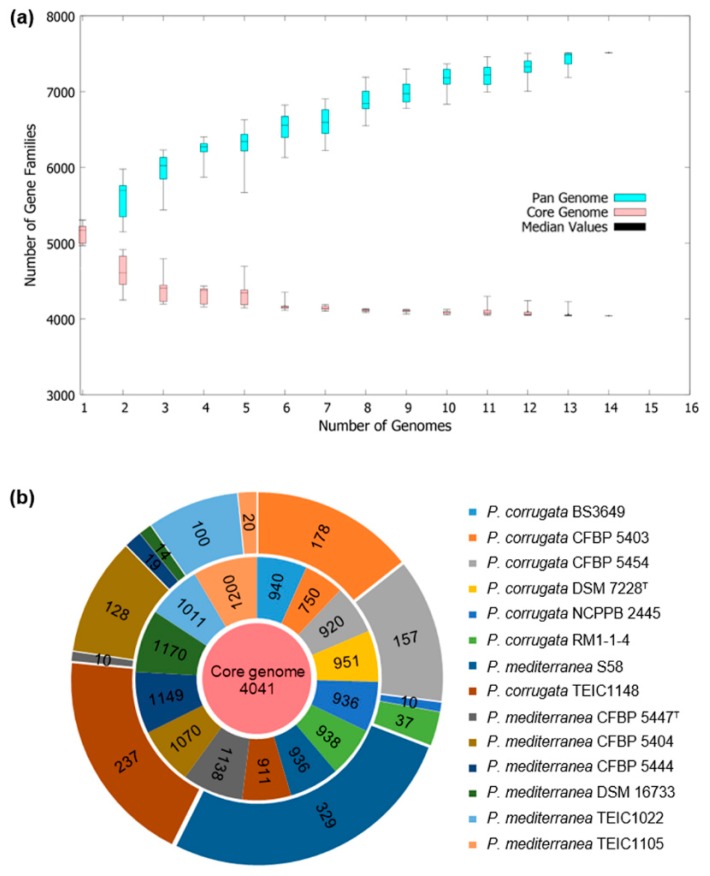
Pan and core genome analysis of the strains of *P. mediterranea* and *P. corrugata*. **(a)** Pan-genome and core genome plot show the progression of the pan (blue line) and core (pink line) genomes as more genomes are added for analysis; **(b)** The flower plot shows the numbers of core genes (inner circle), accessory genes (middle circle), and unique genes (outer circle).

A summary of the general sequence characteristics is given in Table 1. The size of the genome of *P. mediterranea* S58 was 6,150,838 bp with a 61.06% G+C content, and it was predicted to contain 5594 coding sequences (CDSs) that included 5312 genes with an average length of 992.90 bp (Table 1). The total sequence of the CDSs covered 87.62% of the entire genome. In addition, 282 genes were assigned pseudogenes due to a missing N- and C-terminus or a frameshift mutation. A total of 67 tRNA, 15 rRNAs, and four non-coding RNA genes were predicted to occur on the chromosome. A genome circle map of *P. mediterranea* S58 and the closely related three strains (i.e., *P. mediterranea* CFBP 5447^T^ [38], *P. corrugata* DSM 7228^T^ [39], and *P. corrugata* RM1-1-4 [40]) was generated using BRIG (Figure S4). Conservation and variation in gene content between genomes were visualized from the image. It should be noted that the CFBP 5447^T^ and DSM 7228^T^ genomes are draft genomes, and only the genome of RM1-1-4 was closed completely. Therefore, regions that were not included in the BRIG alignment most likely represented regions not sequenced in one or more genomes. To identify phylogenomics and orthologous genes common to these two related species, we carried out a pan-genome and core genome analysis of all 13 strains included in *P. mediterranea* and *P. corrugata* (Figure 7). The pan-genome of all the strains had 18,446 genes, which was the sum of the core genes and dispensable genes, which included unique genes and accessory genes (Figure 7a). Of the identified genes, 4041 were core genes and 329 were unique to strain S58 (Figure 7b).

Given the versatile plant growth-promoting traits of *P. mediterranea* S58, the potential to produce secondary metabolites was analyzed using the online pipeline antiSMASH 5.0. Ten putative gene clusters involved in the biosynthesis of different natural products were found, which included seven nonribosomal peptide-synthetase (NRPS) clusters that encoded antimicrobial cyclic lipopeptides (CLPs) (Table 2). Moreover, one bacteriocin biosynthetic gene cluster was found. No polyketide-encoding gene clusters were predicted. Interestingly, two clusters that encoded crochelin A and Orfamide B were present only in *P. mediterranea* S58, but not in *P. corrugata* RM1-1-4, which was the only complete sequenced strain in the *P. mediterranea* and *P. corrugata* group (Table 2). A *pqqFABDE* operon (E3Z27_25820- E3Z27_25845) was present, which might be involved in gluconic acid (GA) biosynthesis used for solubilization of phosphates [41]. It was tested that *P. mediterranea* S58 could produce plant growth hormone IAA. A high identity (89.94%) homolog of aldehyde dehydrogenase (AldA) was found in the genome, which was reported as a part of a novel IAA synthesis pathway in *P. syringae* strain DC3000 [42]. Further genome analysis revealed that *P. mediterranea* S58 contained genes related to other plant growth-promoting traits, such as the production of siderophores, glutamine synthetase, protease, chitinase, and 1-aminocyclopropane-1-carboxylic acid deaminase.

### 3.6. Strain S58 Triggered Cell Death and Plant Immunity

**Figure 8:**
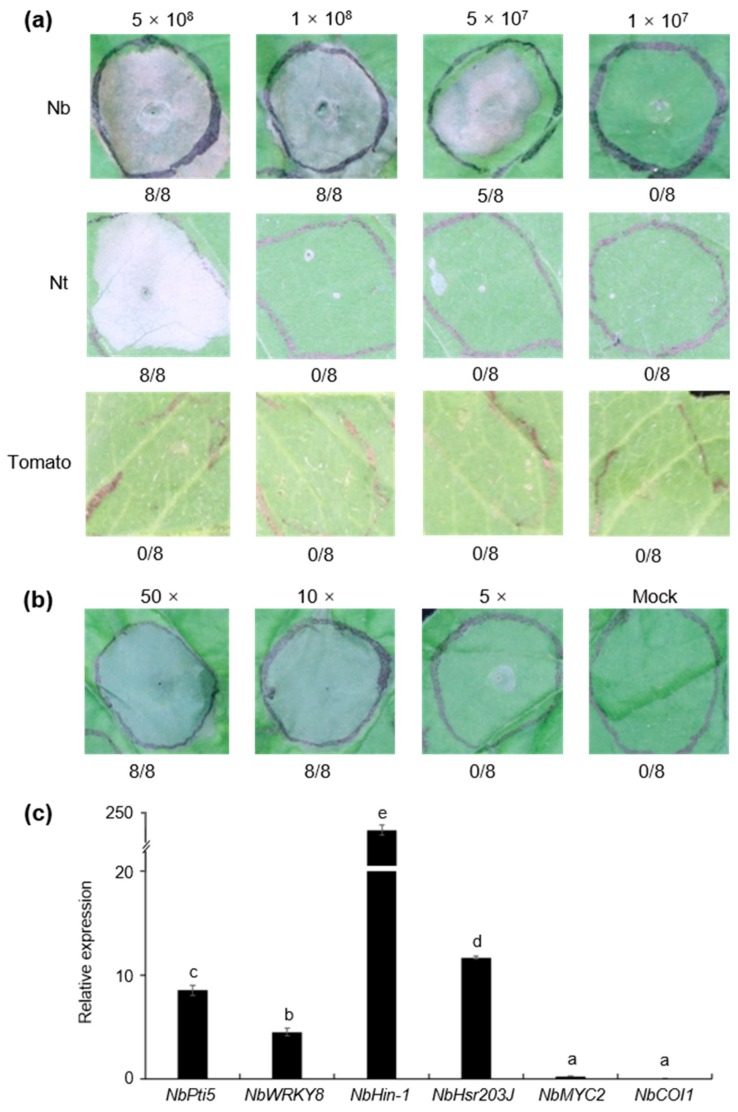
Cell death-like immunity triggered by *P. mediterranea* S58. (**a**) Cell death assays with strain S58 at a concentration gradient on different plants. The fraction under each image indicates the number of times that the cell death occurred relative to the number of test inoculations. Nb, Nt, and tomato represent *N. benthamiana*, *Nicotiana tabacum*,and *L. esculentum* cv. Moneymaker, respectively; (**b**) Cell death assays with the supernatant of strain S58 at different concentrations in *N. benthamiana*; (**c**) Expression of immunity marker genes was tested by qRT-PCR. Relative expression levels were normalized to the reference gene *NbEF1α*. Means with the same letters are not different statistically based on a Tukey test (α = 0.05).

A test of hypersensitive response (HR)-like cell death was performed by infiltrating 5 × 10^8^ CFU/mL of bacteria into the leaves of *Nicotiana benthamiana*. Significant necrosis was present at 24 h post-inoculation (Figure 8a). The titer was determined for the lowest concentration of *P. mediterranea* S58 to trigger cell death. The results showed that the threshold concentration for cell death was 5 × 10^7^ CFU/mL (Figure 8a). We determined further whether *P. mediterranea* S58 triggered cell death in N. tabacum and tomato (*Lycopersicon esculentum* cv. Moneymaker). Interestingly, *P. mediterranea* S58 only induced cell death in *N. tabacum* at a high concentration of infiltrate of 5 × 10^8^ CFU/mL but failed to elicit any cell death-like symptoms in tomato at any of the tested gradients (Figure 8a). We then asked whether the cell death was induced by extracellular components of *P. mediterranea* S58. The concentrated supernatant of the bacterial culture was tested for cell death, in which tenfold and fivefold concentrations elicited visible cell death (Figure 8b).

Cell death is a process by which the plants produce immune responses to restrict microbial infection. A series of gene markers was selected to determine immunity using qRT-PCR. The expressions of NbPti5 and NbWRK7, which are two pattern-triggered immunity (PTI) marker genes that are generally induced by the flagellin protein and its flg22 peptide epitope, were up-regulated by *P. mediterranea* S58 at 12 dpi. This suggested that the flagellin protein of the *P. mediterranea* S58 might be a Microbe-Associated Molecular Pattern (MAMP) to trigger PTI. Unsurprisingly, NbHin-1 and NbHsr203J, which are two cell death marker genes, were up-regulated sharply by *P. mediterranea* S58. The expression of cell death markers was significantly higher than PTI markers. However, the jasmonic acid (JA) signaling genes (NbMYC2 and NbCOI1) that were involved in rhizobacteria-mediated Induced Systemic Resistance (ISR) were not affected at all by *P. mediterranea* S58. All these results suggested that *P. mediterranea* S58 triggered potent, cell death-like, local innate immunity in tobacco.

## 4. Discussion

Pseudomonas spp. are major plant-associated bacteria that occupy different niches and exhibit varied effects on plants [2]. They can act as pathogens, such as *P. syringae* that cause diseases in most higher plants, or they can act as plant growth-promoting agents, such as *P. fluorescens* that controls plant fungal and bacterial diseases [4]. Some *P. mediterranea* strains, a weak and opportunistic pathogen, were isolated in association with tomato pith necrosis [18]. In addition, a number of *P. mediterranea* strains have been isolated from non-diseased plants, mainly from rhizospheres and roots. These strains showed multiple beneficial traits in plants, such as the production of antimicrobial products and plant hormones [39]. S58 is one strain of *P. mediterranea* that was isolated from the tobacco rhizosphere. In a comprehensive in vitro test, it inhibited the growth of a wide range of plant pathogens and produced siderophores, protease, ammonia, and indole-3-acetic acid. It also controlled wheat and tobacco diseases efficiently, promoted the development of *Arabidopsis thaliana*, and induced cell death-like immunity. All of those results suggested that *P. mediterranea* S58 is a potent PGPR with versatile beneficial characters.

It is noteworthy that Pseudomonas species or strains are closely related, but their identification and taxonomy cannot be defined simply by relying on a single approach [39]. *P. mediterranea* is a member of the P. corrugata subgroup of the P. fluorescens group based on MLSA studies that used the 16S rRNA gene and *rpoB, rpoD*, and *gyrB* gene sequences [43,44]. In this study, we performed morphological, biochemical, physiological characterization, and MLSA phylogenic analysis. The results distinctly showed that strain S58 belonged in the species *P. mediterranea*. Further analysis of the ANI values of the phylogenetically related species and strains also revealed that strain S58 belonged to *P. mediterranea*. Although it has been almost two decades since *P. mediterranea* was identified as a new species, only six strains had been sequenced prior to this study. However, all of the released sequences are draft genomes, which indicates that we have only a superficial understanding of the molecular mechanisms; this restricts the application of *P. mediterranea* as bioderemediators or PGPR.

The complete genome of *P. mediterranea* S58 provides insights into the genes involved in rhizo competence. The core genome of 13 genome sequences from *P. mediterranea* and *P. corrugata* was composed of 4041 orthologous genes, which is larger in comparison with a previous analysis that used 10 representative genome sequences of the *P. fluorescens* group (2789 orthologous genes) [44]. In addition, 1265 dispensable genes (including 329 unique and 936 accessory genes) that we found in the S58 genome are not present in any other genomes of strains within this comparison. The most dispensable genes in *P. mediterranea* S58 suggests that it has more specific and versatile metabolisms. We need to note that strain S58 triggered cell death in tobacco (Figure 8a), which was confirmed by up-regulated expressions of NbHin-1 and NbHsr203J, two typical cell death marker genes (Figure 8c). The pathogen *P. syringae* induced type III secretion system (T3SS)-dependent cell death in non-host plants [45]. Some T3SS effector and Harpin proteins contributed to cell death [45]. An in-depth investigation of the proteomes of *P. mediterranea* S58 using all T3SS components revealed that it did not contain any T3SS homologs that included effector and Harpin proteins, which are identical to the findings in other *P. corrugata* and *P. mediterranea* isolates [39]. This suggested that other secreted proteins, phytotoxins, or secondary metabolites, but not T3SS-related proteins, conferred the phenotype with cell death capability. Syringotoxin and syringomycin E isloated from *P. syringae* triggered cell death in tobacco [7]. Recently, cormycin A, a cyclic lipodepsipeptide (CLP) isolated from the culture of *P. corrugata* NCPPB 2445, also induced cell death in tobacco [46]. It is a CLP-rich bacterium; hence, *P. mediterranea* S58 might rely on some CLPs to induce hypersensitive cell death in plants. Our study opens up opportunities for the exploitation of the molecular mechanisms of the versatile PGPR traits in *P. mediterranea* S58 and related species.

## 5. Patents

The work reported in the manuscript has been granted a Chinese invention patent (ZL201910911436).

## Figures and Tables

**Table 1:** General features of the *P. mediterranea* S58 genome.

Features	Chromosome
Size (bp)	6,486,667
G+C content (%)	61.06
Number of total CDSs	5594
Number of genes	5312
Pseudogenes	282
tRNAs	67
rRNA genes	15
ncRNAs	4
Contigs	1
Total CDSs size (bp)	5,683,341
Coding %	87.62
Average CDS length (nt)	992.90

**Table 2:** List of predicted secondary metabolite biosynthetic gene clusters of *P. mediterranea* S58.

Cluster Type/Metabolites	S58		RM1-1-4 ^1^
	Location	Gene ID	
NRPS-like/Fragin	177591-219516	E3Z27_00825-E3Z27_00960	+
Arylpolyene/APE Vf	476416-520027	E3Z27_02080-E3Z27_02290	+
Bacteriocin	1422090-1432977	E3Z27_06235-E3Z27_06295	+
NAGGN	1834296-1849084	E3Z27_08105-E3Z27_08160	+
NRPS/Fengycin	2221816-2245051	E3Z27_09835-E3Z27_-09940	+
NRPS/Crochelin A	2691862-2766373	E3Z27_12005-E3Z27_12180	-
NRPS-like/Entolysin	2967333-3015201	E3Z27_12950-E3Z27_13125	+
NRPS/Orfamide B	3271309-3337076	E3Z27_14180-E3Z27_14400	-
NRPS/Siderophore	3444389-3463297	E3Z27_14930-E3Z27_15000	+
NRPS/Syringomycin	3672567-3850583	E3Z27_15910-E3Z27_16205	+

^1^ represents *P. corrugata* RM1-1-4.

## Supplementary Materials

The following are available online at https://www.mdpi.com/2076-2607/8/3/334/s1, Figure S1: Symptoms of tobacco wildfire disease, Figure S2: No antagonism of strain S58 against *Pseudomonas syringae* pv. *tabaci*, Figure S3: Electron micrograph of the cells of strain S58, Figure S4: Genome comparison of strain S58, *Pseudomonas corrugata*, and *Pseudomonas mediterranea*. The circles from inner to external: GC Content, GC Skew, *P. mediterranea* S58 (pink), *P. mediterranea* CFBP 5447^T^ (green), *P. corrugata* DSM 7228^T^ (blue), and *P. corrugata* RM1-1-4 (light blue), Table S1: Sequences of the gene-specific primer pairs used in the study, Table S2: Physiological and biochemical indexes of strain S58.
