# Supplementary material for: Characterization of a Versatile Plant Growth-Promoting Rhizobacterium Pseudomonas mediterranea Strain S58
Source: Microorganisms. 2020 Feb 27;8(3):334. doi: 10.3390/microorganisms8030334 (PMC7143339; doi:10.3390/microorganisms8030334)
Supplement: Supplementary file 1 [file microorganisms-08-00334-s001.zip › Supplementary files/Table S1 and S2.docx]

**Table S1.** Sequences of the gene-specific primer pairs used in the study

| **Primer name** | **Primer sequence** | **Reference** |
| --- | --- | --- |
| NbPti5-F | CCTCCAAGTTTGAGCTCGGATAGT | [1] |
| NbPti5-R | CCAAGAAATTCTCCATGCACTCTGTC |  |
| NbWRKY8-F | AACAATGGTGCCAATAATGC | [1] |
| NbWRKY8-R | TGCATATCCTGAGAAACCATT |  |
| NbHin1-F | AGTTGTCTCTTTGGATGCCTCTGC | [2] |
| NbHin1-R | ACTGAGTCAACGTAGCATCGGTCA |  |
| NbHsr203J-F | CGCAATTCCAATCCATCCAGGCTT | [2] |
| NbHsr203J-R | GCAATTTAAGCTCCTCAACCGCCT |  |
| NbPR1a-RT-F | GTAATATCCCACTCTTGCCG | [3] |
| NbPR1a-RT-R | ATGAAATCGCCACTTCCCTC |  |
| NbNPR1-RT-F | TTACTTCACTGAAACGCCT | [3] |
| NbNPR1-RT-R | CACTTCCTTTAATTCCACCT |  |
| NbMYC2-F | GAAGCGGATAGTAGTAGAGTT | [4] |
| NbMYC2-R | TTTCTCCCTCCTTTGTCT |  |
| NbCOI1-F | GAACAGGAAATGGAGGAC | [4] |
| NbCOI1-R | AGTAGAACCAACCGAAAA |  |
| NbEF1a-F | TGGACACAGGGACTTCATCA | [5] |
| NbEF1a-R | CAAGGGTGAAAGCAAGCAAT |  |

1. Mclellan, H.; Boevink, P.C.; Armstrong, M.R.; Pritchard, L.; Gomez, S.; Morales, J.; Whisson, S.C.; Beynon, J.L.; Birch, P.R.J. An RxLR Effector from *Phytophthora infestans* prevents re-localisation of two plant *NAC* transcription factors from the endoplasmic reticulum to the nucleus. *PLoS Pathog.* 2013, 9(10):e1003670. doi: 10.1371/journal.ppat.1003670
2. Wei, H.L.; Chakravarthy, S.; Worley, J.N; Collmer, A. Consequences of flagellin export through the type III secretion system of *Pseudomonas syringae* reveal a major difference in the innate immune systems of mammals and the model plant *Nicotiana benthamiana*. *Cell. Microbiol.* 2013, 15(4), 601-618.
3. Zhang, H.X.; Ali, M.; Feng, X.H.; Jin, J.H.; Huang, L.J.; Khan, A.; Lv, J.G.; Gao, S.Y.; Luo, D.X.; Gong, Z.H. A novel transcription factor CaSBP12 gene negatively regulates the defense response against *Phytophthora capsici* in pepper (*Capsicum annuum* L.). *Int. J. Mol. Sci.* 2018, doi: 10.3390/ijms20010048
4. Yan, Q.; Cui, X.; Lin, S.; Gan, S.; Xing, H.; Dou, D. GmCYP82A3, a soybean cytochrome P450 family gene involved in the jasmonic acid and ethylene signaling pathway, enhances plant resistance to biotic and abiotic stresses. *PLoS ONE*, 2016, 11(9):e0162253. doi: 10.1371/journal.pone.0162253

**Table S2.** Physiological and biochemical indexes of strain S58

| **Indexes** | **Results** | **Indexes** | **Results** | **Indexes** | **Results** |
| --- | --- | --- | --- | --- | --- |
| Negative control | - ^1^ | Inosine | - | D-glucuronic acid | W |
| Dextrin | - | 1% Na-lactate | + | Glucuronamide | - |
| D-maltose | - | Fusidic acid | - | Mucate | + |
| D-trehalose | + ^2^ | D-serine | - | Quinate | + |
| D-cellobiose | - | D-sorbitol | - | D-saccharate | + |
| Gentiobiose | - | D-mannitol | + | Vancomycin | + |
| Sucrose | + | D-arabitol | - | Tetrazolium violet | + |
| D-turanose | - | myo-inositol | W | Tetrazolium blue | + |
| N-acetyl-D-glucosamine | - | D-lactic acid methyl ester | - | p-hydroxy-phenylacetic acid | + |
| Positive control | + | D-glucose-6-PO4 | - | Methyl pyruvate | W |
| pH 6 | + | D-fructose-6-PO4 | W | Glycerol | - |
| pH 5 | - | D-aspartic acid | W | L-lactic acid | + |
| D-raffinose | - | D-serine | - | Citric acid | + |
| α-D-lactose | - | Troleandomycin | + | α-keto-glutaric acid | + |
| D-melibiose | - | Rifamycin SV | + | D-malate | - |
| β-methyl-D-glucoside | - | Minocycline | - | L-malate | + |
| D-salicin | - | Gelatin | - | Bromo-succininate | - |
| Stachyose | - | Glycyl-L-proline | - | Nalidixic acid | + |
| K-tellurite | + | L-alanine | + | LiCl | - |
| N-acetyl-D-galactosamine | - | β-hydroxy-D,L-butyric acid | - | N-acetyl-β-D-mannosamine | - |
| N-acetyl neuraminic acid | - | L-galactonic acid lactone | - | α-hydroxy-butyric acid | - |
| 1% NaCl | + | L-glutamate | + | aminobutyrate | + |
| 4% NaCl | - | L-aspartic acid | W | Tween-40 | - |
| 8% NaCl | - | L-pyroglutamic acid | + | L-arginine | - |
| D-glucose | + | L-serine | - | α-keto-butyric acid | - |
| D-mannose | + | Lincomycin | + | Acetoacetic acid | - |
| D-fructose | + | Guanidine HCl | + | Propionic acid | W |
| D-galactose | + | Niaproof 4 | + | Acetic acid | + |
| 3-methyl glucose | - | Pectin | + | Formic acid | - |
| D-fucose | W ^3^ | D-galacturonic acid | W | Aztreonam | + |
| L-fucose | - | L-histidine | - | Sodium butyrate | - |
| L-rhamnose | - | D-gluconate | + | Sodium bromate | - |

^1^ means negative; ^2^ means positive; ^3^ means weak positive
