# Supplementary figures and images for: Characterization of a Versatile Plant Growth-Promoting Rhizobacterium Pseudomonas mediterranea Strain S58
Source: Microorganisms. 2020 Feb 27;8(3):334. doi: 10.3390/microorganisms8030334 (PMC7143339; doi:10.3390/microorganisms8030334)

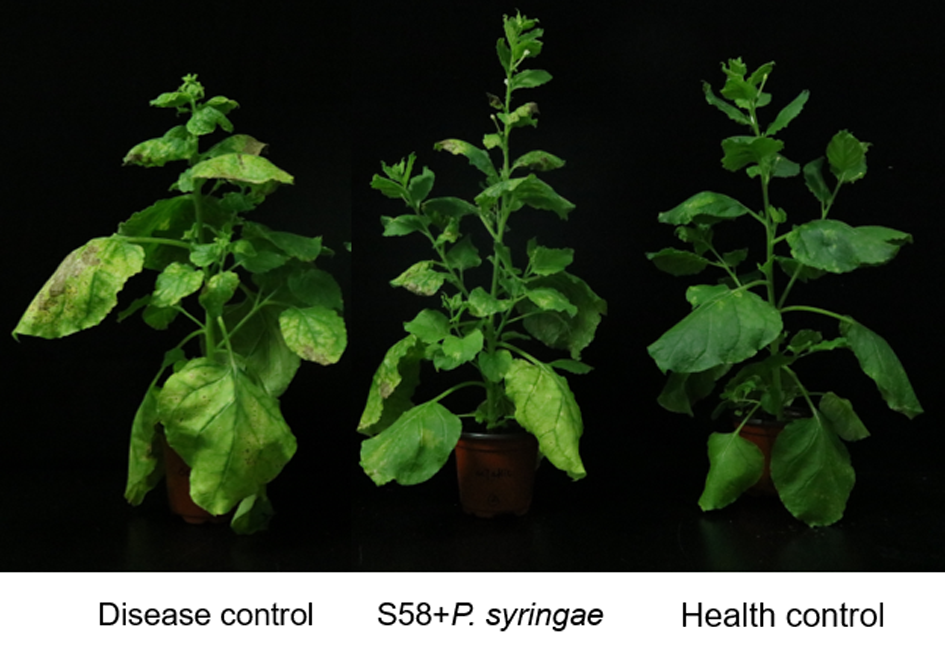

Supplement: Supplementary file 1 [file microorganisms-08-00334-s001.zip › Supplementary files/Figure S1.tif]

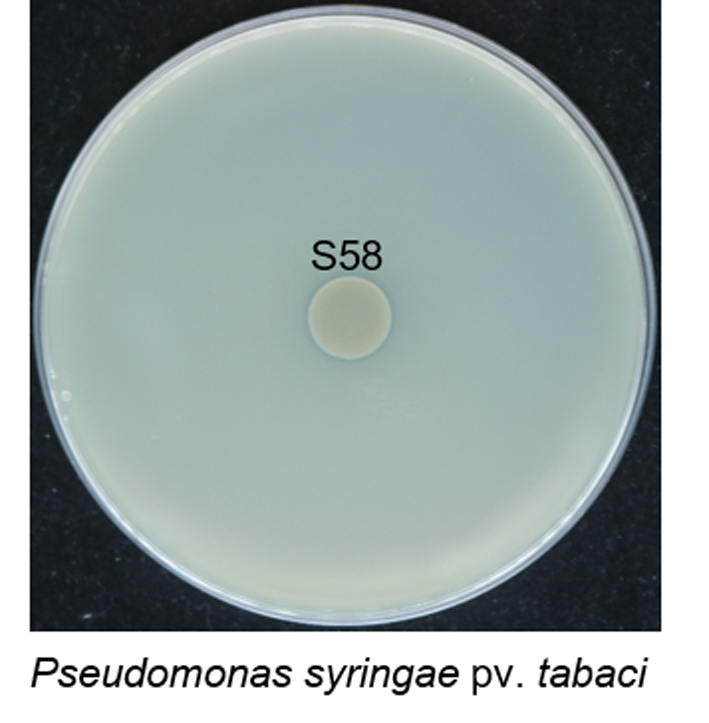

Supplement: Supplementary file 1 [file microorganisms-08-00334-s001.zip › Supplementary files/Figure S2.tif]

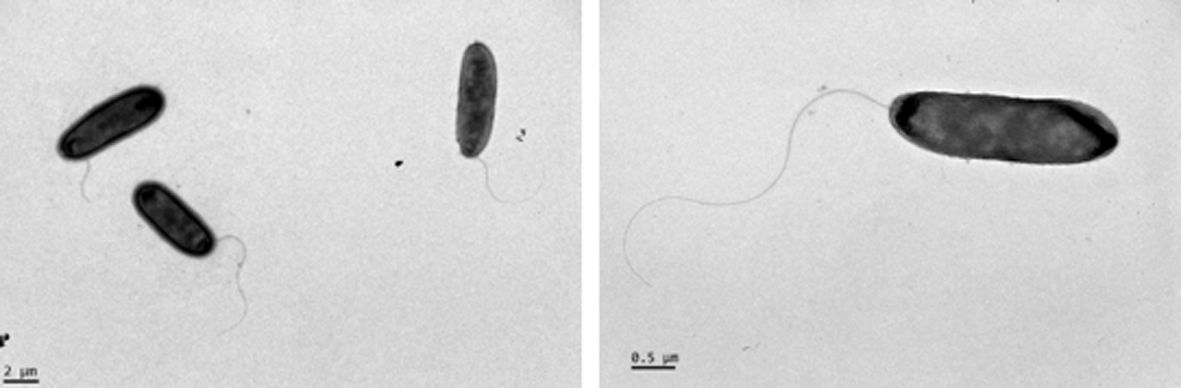

Supplement: Supplementary file 1 [file microorganisms-08-00334-s001.zip › Supplementary files/Figure S3.tif]

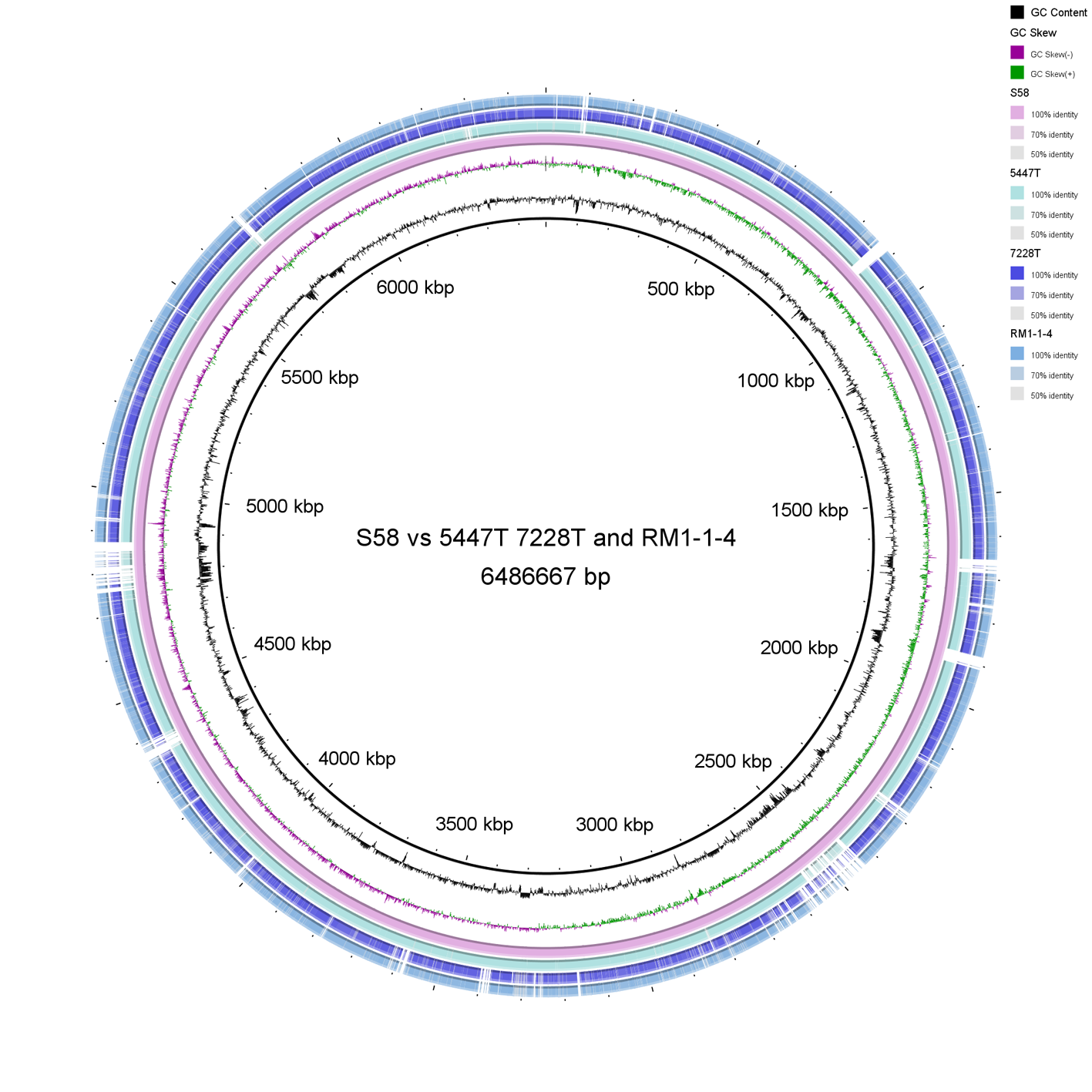

Supplement: Supplementary file 1 [file microorganisms-08-00334-s001.zip › Supplementary files/Figure S4.tif]
